# The role of the superior temporal lobe in auditory false perceptions: A transcranial direct current stimulation study

**DOI:** 10.1016/j.neuropsychologia.2014.07.032

**Published:** 2014-09

**Authors:** Peter Moseley, Charles Fernyhough, Amanda Ellison

**Affiliations:** Psychology Department, Durham University, South Road, Durham DH1 3LE, UK

**Keywords:** Auditory verbal hallucinations, Signal detection, Superior temporal gyrus, tDCS

## Abstract

Neuroimaging has shown that a network of cortical areas, which includes the superior temporal gyrus, is active during auditory verbal hallucinations (AVHs). In the present study, healthy, non-hallucinating participants (*N*=30) completed an auditory signal detection task, in which participants were required to detect a voice in short bursts of white noise, with the variable of interest being the rate of false auditory verbal perceptions. This paradigm was coupled with transcranial direct current stimulation, a noninvasive brain stimulation technique, to test the involvement of the left posterior superior temporal gyrus in the creation of auditory false perceptions. The results showed that increasing the levels of excitability in this region led to a higher rate of ‘false alarm’ responses than when levels of excitability were decreased, with false alarm responses under a sham stimulation condition lying at a mid-point between anodal and cathodal stimulation conditions. There were also corresponding changes in signal detection parameters. These results are discussed in terms of prominent cognitive neuroscientific theories of AVHs, and potential future directions for research are outlined.

## Highlights

• Investigated role of left STG in false perceptions of voices in white noise.
• Used noninvasive brain stimulation whilst participants listened to white noise.
• Tested whether number of false perceptions was affected by stimulation of STG.
• Higher false alarm rate when excitability increased than when decreased.

## 1 Introduction

Auditory verbal hallucinations (AVHs) are the experience of hearing a voice in the absence of any speaker. Although experienced by between 60% and 80% of people with a diagnosis of schizophrenia (Sartorius et al., 1986), the experience is also reported by approximately 1.5–3% of the general population (Beavan et al., 2011, Tien, 1991). Neuroimaging findings relating to AVHs have been variable, but tend to show that AVHs coincide with activation in areas of the temporal lobe such as the superior temporal gyrus (STG), and frontal lobe areas such as the inferior frontal gyrus (IFG) and anterior cingulate cortex (ACC) (Allen et al., 2012).

The STG encompasses primary auditory cortex (PAC), as well as secondary auditory cortices such as Wernicke׳s area/the temporoparietal junction (TPJ), and the planum temporale (PT). Due to its importance in auditory processing, the role of the STG in AVHs (and associated cognitive mechanisms), particularly in the left hemisphere, has been extensively studied. For example, repeated measurements have shown tonic hyperactivity in left STG in patients with a diagnosis of schizophrenia who experience AVHs (Homan et al., 2013). Meta-analytic findings show that, in people who experience AVHs, PAC shows reduced activation to external auditory stimuli, but increased activation to internally generated information such as AVHs (Kompus, Westerhausen & Hugdahl, 2011). In addition, patients with a diagnosis of schizophrenia show reduced attenuation in auditory cortex when using inner speech (Simons et al., 2010), and reduced attenuation in somatosensory cortex when experiencing tactile stimulation (Shergill et al., 2014). These findings may reflect failures of internal forward models to successfully attenuate activity in response to self-produced actions (Ford & Mathalon, 2005), and/or biased attentional processes (Kompus et al., 2011). Finally, using offline repetitive transcranial magnetic stimulation (rTMS) or transcranial direct current stimulation (tDCS) to decrease activity in Wernicke׳s area (left posterior STG) as a treatment protocol has been shown to reduce the frequency of AVHs (Brunelin et al., 2012, Hoffman et al., 2013, Slotema et al., 2013), possibly due to effects on activity in other auditory cortical areas in the left STG (Kindler et al., 2013).

The above evidence suggests that the left pSTG plays a crucial role in the generation and/or experience of AVHs. This is in concordance with neuroimaging evidence suggesting that, among other areas, the superior temporal gyrus is active in the neurotypical brain during verbal self-monitoring (Allen et al., 2007, McGuire et al., 1995), and when a voice is falsely detected in white noise (Barkus, Stirling, Hopkins, McKie & Lewis, 2007), an error that people who experience AVHs make more often (Brookwell, Bentall & Varese, 2013). Nevertheless, the majority of available evidence regarding the role of the STG comes from fMRI and, due to the inherently correlational nature of neuroimaging, it is hard to draw conclusions about the causality of the role of this brain area in AVHs.

Whilst attempts to treat AVHs using neurostimulation of STG or TPJ are suggestive of the critical importance of these regions, and of surrounding auditory cortical areas (Kindler et al., 2013, Moseley et al., 2013 ), it remains to be determined how neural activations relate to underlying cognitive mechanisms. For example, if the STG is causally involved in the genesis of AVHs, it should be possible to both increase and decrease AVH frequency by modulating the level of activity accordingly. Whilst this is clearly not possible in a clinical sample due to ethical issues, one previous approach has been to use a signal detection task, in which healthy participants are asked to listen to bursts of white noise, and respond whether they believe a voice is present (Bentall & Slade, 1985). This approach enables an analysis of ‘correct’ perceptions, as well as ‘false’ perceptions (or ‘false alarm’ responses).

Previous research suggests that individuals with a diagnosis of schizophrenia who hallucinate, and non-clinical participants who report more frequent hallucinatory experiences, are more likely to falsely perceive a voice in the noise (Barkus et al., 2011, Brookwell et al., 2013, Varese et al., 2011). These studies employ signal detection analysis, and suggest that this finding is due to a difference in response bias (i.e., how willing participants are to accept that an ambiguous stimuli is present) between hallucinators and non-hallucinators, rather than a change in sensitivity to the task (the ability to distinguish between signal and noise). This is important, as it implies that individuals who experience AVHs do not have a ‘deficit’ on the task, but instead simply exhibit a different style of responding. However, in a study by Vercammen, de Haan and Aleman (2008) using a similar paradigm, participants who experienced AVHs showed both a lower response bias *and* lowered sensitivity to the task, suggesting that the group differences may be more complex than a response bias. Of equal importance, false perceptions on this task are associated with high levels of activation in, among other areas, the STG (Barkus et al., 2007), even compared to correct perceptions of a voice in the noise. This suggests that high levels of activity in the STG might be associated with false alarm responses in this task, perhaps reflecting a tendency to misattribute internal, self-generated processes to an external source, as in AVHs.

Nevertheless, as discussed, evidence that activity in the STG is the *cause* of false alarm responses in a signal detection task is lacking. To address this, we utilised a form of non-invasive brain stimulation, transcranial direct current stimulation (tDCS), to modulate excitability in the left posterior STG (pSTG) of non-clinical, non-hallucinating participants. tDCS involves running a weak electrical current between two electrodes in contact with the participant׳s scalp, depolarising (anodal) or hyperpolarising (cathodal) membrane potentials of underlying neurons, resulting in a decrease in potential activity under the cathode and an increase in potential activity under the anode (Nitsche & Paulus, 2000). Furthermore, once stimulation has stopped, a reduction in GABA concentration under the anodal electrode and glutamate concentration under the cathodal electrode can be observed (Stagg & Nitsche, 2011), as well as short-lasting behavioural effects (Hummel & Cohen, 2006).

There are two main advantages of using non-clinical samples to study hallucination-like experiences:1) results are not confounded by anti-psychotic medication or additional symptoms of psychosis; 2) it would not be ethical to attempt to increase cortical excitability in a population which may already experience potentially pathological over-activity in superior temporal regions. Our objective was to test whether modulating excitability in left pSTG would lead to a change in the number of false perceptions that participants would make on an auditory signal detection task. Specifically, given findings that levels of activity in this region are related to both AVHs and false perceptions on auditory signal detection, we hypothesised that increasing the excitability of the posterior STG using anodal stimulation would lead to an increase in false alarms, whereas decreasing excitability using cathodal stimulation would lead to a decrease in the number of false alarms.

## 2 Materials and methods

### 2.1 Participants

The sample consisted of 30 right-handed participants (7 males, 23 females), aged 18–26 (*M*=20.6, SD=2.67). Participants were considered ineligible to take part if they reported any hearing problems, or any history of neurological or psychiatric disorder. All gave written informed consent in accordance with the Declaration of Helsinki, and ethical approval was provided by Durham University Ethics Committee. Participants were paid £15 for participation, and were naive to the aim of the study, simply being told that the study was investigating ‘auditory perception’.

### 2.2 Signal detection task

The stimuli used in the signal detection task were similar to those used by Barkus et al., 2007, Barkus et al., 2011, in which participants were asked to detect a voice stimulus embedded in white noise. The voice stimuli were identical to those used by Barkus et al.; a neutral, androgynous voice reading text from an instruction manual, which was segmented into 1-s clips. To set the volume levels in the task, we ran a small pilot study (*N*=8, none of whom took part in the main study), in which participants listened to a continuous burst of white noise, within which the voice clips were played, at a gradually ascending volume level. Participants were simply asked to respond with a button press when they heard a voice, and each pilot participant׳s threshold was defined as the point at which they heard three consecutive voices. For the main task, we then set the volume levels at the point at which 100%, 75%, 50% and 25% of participants in the pilot study consistently detected the voices (henceforth referred to as volume levels 4, 3, 2 and 1, respectively).

The stimuli for the main task consisted of 144 5-s bursts of white noise. During 80 bursts, a voice was present for the middle 1 s (‘voice-present’ trials). In the voice-present trials, voices were played at one of the four volume levels, which were kept constant across all participants (a requirement of the analysis, based on signal detection theory). The remaining 64 ‘voice-absent’ trials consisted of the white noise, with no embedded voice. Each burst was followed by 3 s of silence, in which the participant was instructed to respond with a button press whether they believed a voice was present in the noise (yes/no). The stimuli were pseudo-randomly ordered, so that none of the five possible trial types (voice-absent, plus four voice-present volume levels) was presented more than three times in a row. Participants were not informed how often a voice was likely to be present, but were told that voices may be present at a variety of volumes. The task was separated into two blocks, each lasting 576 s, with a 5  min break between the blocks.

### 2.3 Transcranial direct current stimulation

Participants received 15 min of tDCS, using a Magstim Eldith DC stimulator. A 1.5 mA current was delivered to the first 14 participants, but for the final 16 participants this was decreased to 1 mA, after two participants experienced a mild headache following stimulation. The current was delivered through rubber electrodes placed in saline-soaked sponges, held in place by two rubber straps. One electrode (5×5 cm=25 cm^2^) was positioned over the left posterior superior temporal gyrus (pSTG), over electrode site CP5 according to the EEG 10–20 system. This system ensures that the electrode montage is adjusted for differing head sizes between participants, and has been used previously to target the superior temporal gyrus, and more specifically, Wernicke׳s area (You, Kim, Chun, Jung & Park, 2011). The second electrode (5×7 cm=35 cm^2^) was positioned above the right eye, as in other tDCS studies (Ball et al., 2013, Ellison et al., 2014). A contralateral location was chosen as this is the most commonly used in the tDCS literature (Nitsche et al., 2008). The difference in electrode size ensured that the stimulation under the superior temporal electrode reached a higher current density than under the larger electrode. There were three stimulation conditions over the pSTG: anodal, cathodal and sham stimulation. Each participant received each type of stimulation in separate sessions, with each session separated, where possible, by 7 days (mean no. days between Sessions 1–2=7.47, SD=1.55, range=6–14; mean no. days between Sessions 2–3=7.80, SD=2.51, range=3–14). The order in which participants received the three types of stimulation was counterbalanced, so that all six possible orders were represented equally in the sample. Anodal and cathodal stimulations of the pSTG consisted of 900 s (15 min) of stimulation, plus 8 s during which the strength of the stimulation gradually faded in, and 8 s during which it faded out. Sham stimulation consisted of the application of 30 s of stimulation, plus 8 s fade-in and 8 s fade-out; this method of sham stimulation ensured that the participant experienced the initial tingling sensation on the scalp associated with active stimulation, but did not receive sufficient stimulation to modulate neuronal excitability. This has been demonstrated to be an effective method of blinding participants to the stimulation condition (Gandiga, Hummel & Cohen, 2006).

### 2.4 Procedure

In each session, participants were seated in front of a laptop computer, and were provided with noise-cancelling earbuds (Creative EP-630), through which the stimuli were played. Pilot testing indicated that some participants preferred to close their eyes whilst completing the task; therefore, all participants were blindfolded to prevent between-participant differences in visual input. Participants completed a short practice trial before receiving tDCS, consisting of 8 bursts of white noise. The first block of signal detection trials commenced 340 s after initiation of the stimulation, thus ensuring that the task ended simultaneously with the stimulation. Participants then sat quietly for a 300 s (5 min) break, in which the electrodes were removed from their scalp, to maximise the participant׳s comfort. They then completed a second block of the signal detection trials. The first block of trials is henceforth referred to as ‘online’ (as it was completed whilst active or sham stimulation was applied), and the second block as ‘offline’ (completed after the electrodes had been removed from the scalp).

### 2.5 Data analysis

Responses were categorised into four types: hits (voice-present trial, ‘yes’ response), misses (voice-present trial, ‘no’ response), correct rejections (voice-absent trial, ‘no’ response) and false alarms (voice-absent trial, ‘yes’ response). These responses are expressed as a ‘hit rate’ (the percentage of voice-present trials on which the participant correctly responded ‘yes’) and a ‘false alarm rate’ (the percentage of voice-absent trials on which the participant incorrectly responded ‘yes’). From these, standard signal detection measures for sensitivity and response bias were calculated for each block of trials completed by the participant. *d*′, a measure of sensitivity to the stimulus, is defined as the difference between standardised hit rate and false alarm rate, with a higher score indicating an increased ability to distinguish signal from noise. *β*, a measure of response bias, is calculated as outlined in Stanislaw and Todorov (1999):

$$\beta =e\left\{\frac{Z{\left(FA\right)}^{2}-\;Z{\left(H\right)}^{2}}{2}\right\},$$

where *Z*(*FA*) corresponds to a standardised false alarm rate, and *Z*(*H*) corresponds to a standardised hit rate. Lower *β* values indicate a more ‘liberal’ response bias (i.e., participants are more likely to accept that a voice is present under ambiguous circumstances).

We used a 3×2×2 mixed model design, with the three stimulation conditions (anodal/cathodal/sham) and two task blocks (online/offline) as within-subjects variables. We also included the two stimulation strengths (1.5 mA/1 mA) as a between-subjects variable, to test whether the alteration in current strength affected any potential main effect. We therefore conducted a mixed model ANOVA, using stimulation condition, task block and stimulation strength as independent variables, and false alarm rate as the dependent variable. This analysis was also conducted with signal detection measures as dependent variables (*d*′, *β*), as well as the hit rate (in which volume level was included as a within-subjects variable). We then performed planned contrasts to investigate specifically how false alarm rate differed between the three conditions, using planned paired *t*-tests.

## 3 Results

### 3.1 Effects of tDCS on false alarm rate

**Fig. 1:**
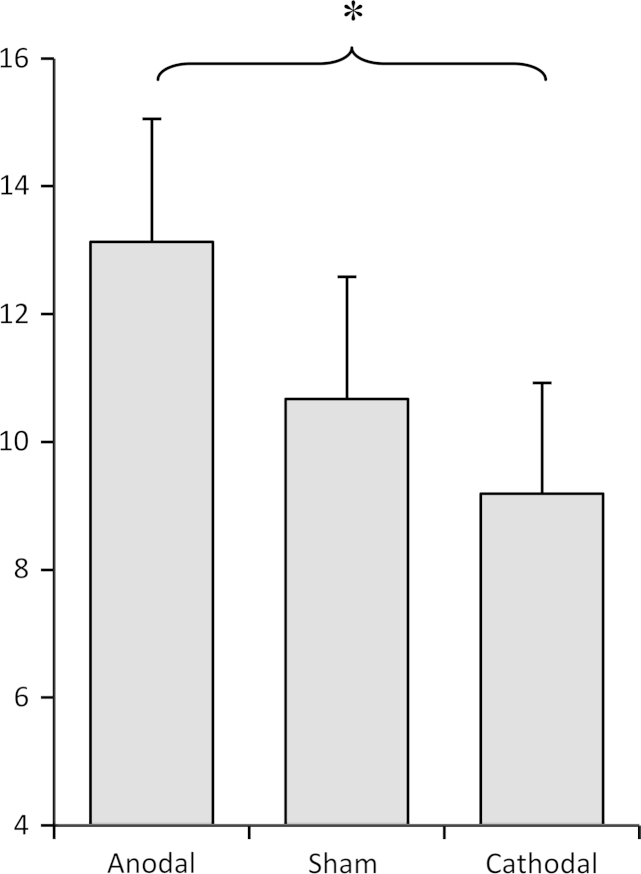
False alarm rate (%) in auditory signal detection task by stimulation condition. Error bars=1 SEM. ^⁎^*p* <.05.

Descriptive statistics for performance on the signal detection task are presented in Table 1. If assumptions of sphericity or homogeneity of variance were not met, then the Greenhouse–Geisser correction was applied. A 3×2×2 (stimulation condition×task block×stimulation strength) mixed model ANOVA showed a significant main effect of stimulation condition on false alarm rate: *F*(2, 56)=3.70, *p* =.031, *η*^2^ =.117. Planned comparisons (one-tailed paired samples *t*-tests) showed that, as predicted, the false alarm rate under the anodal stimulation condition was significantly higher than under the cathodal stimulation condition (*t*(29)=2.52, *p* =.009) (see Fig. 1). However, the difference between the anodal stimulation condition and sham condition only approached significance (*t*(29)=1.54, *p* =.067), and the sham condition did not differ significantly from the cathodal condition (*t*(29)=1.07, *p* =.15). From these results, it is difficult to conclude whether the observed difference in false alarm rate between anodal and cathodal stimulation is due to an effect of one stimulation condition or the other (or both). However, given the similar difference in false alarm rate between anodal/sham and cathodal/sham, it seems probable that the observed effect was due to both an increase in false alarms under the anodal stimulation condition, and a decrease under the cathodal stimulation condition. To back this up, we conducted an exploratory within-subject polynomial contrast analysis, which indicated that there was a significant *linear* trend across the three conditions (*F*(1, 28)=6.42, *p* =.017) suggesting that the false alarm rate varied linearly with the type of stimulation applied.

**Table 1:** Descriptive statistics for hits, false alarms, response bias and sensitivity under each stimulation condition (both task blocks) (*M*, SD). ‘On’ refers to performance during stimulation; ‘Off’ refers to performance five minutes following stimulation. FA=false alarms; *β*=bias; *d*′=sensitivity.

*Type of stimulation*	**Hits (%)**		**FA (%)**		***β***		***d*****′**	
	*On*	*Off*	*On*	*Off*	*On*	*Off*	*On*	*Off*
Anodal	59.17 (11.9)	58.08 (10.9)	15.62 (14.7)	10.64 (10.3)	3.09 (2.5)	4.22 (3.34)	1.45 (.63)	1.64 (.63)
Sham	57.92 (11.9)	56.25 (12.4)	12.05 (11.6)	9.28 (10.6)	4.32 (3.8)	4.99 (3.7)	1.6 (.61)	1.69 (.53)
Cathodal	58.08 (12.4)	58.83 (15.3)	11.69 (11.1)	6.68 (9.1)	3.61 (2.7)	5.88 (4)	1.58 (.5)	1.92 (.5)

There was an effect of task block (online/offline) on false alarm rate: *F*(1, 28)=23.68, *p* <.001, *η*^2^ =.458, implying that false alarm rate tended to drop between the first task block (*M*=13.19, *SD*=10.33) and the second task block (*M*=8.96, SD=8.27). There was no interaction between stimulation condition and task block: *F*(1.6, 43.9) =.396, *p* =.675, *η*^2^ =.014. There was also no interaction between any variables and the strength of the stimulation applied (all *p*s>.15), indicating that the change of current strength between participants did not change the main effect of the stimulation.

### 3.2 Effects of tDCS on other signal detection measures

**Fig. 2:**
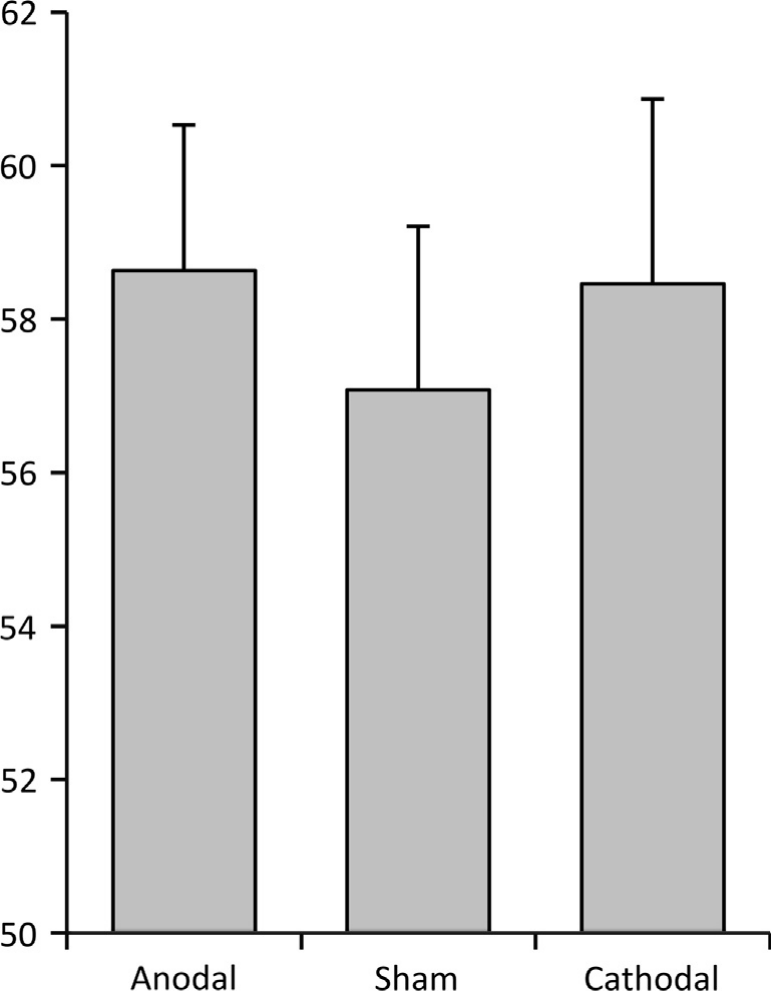
Hit rate (%) in auditory signal detection task by stimulation condition. Error bars=1 SEM.

To explore the effect of stimulation condition on hit rate at differing voice volumes, we conducted a 3×2×2×4 (stimulation condition×block×stimulation strength×voice volume) mixed model ANOVA. As would be expected, there was a significant effect of volume (*F*(2.3, 64.5)=361.20, *p*<.001) on hit rate, showing that participants were more likely to correctly identify voices at higher volumes (see Table 2 for descriptive statistics). There was no effect of tDCS (*F*(2, 56) =.549, *p* =.581) or task block (*F*(1, 28) =.715, *p* =.405) on hit rate, nor any interaction between stimulation condition and task block (*F*(2, 56) =.581, *p* =.563) (see Fig. 2). There was no interaction between voice volume and any variables (all *p*s >.097) or between stimulation strength and any variables (all *p*s >.49) From a signal detection perspective, a decrease in response bias (*β*) would predict an increase in false alarm rate (as observed), but also a corresponding increase in hit rate, especially in the voice stimuli presented at low volumes. Unexpectedly, our results do not support this hypothesis, since there was no effect of stimulation on overall hit rate; that is, stimulation condition affected the number of false perceptions that participants made, but not the number of correct perceptions. There was also no interaction between voice volume and stimulation condition (*F*(6, 174) =.450, *p* =.844), indicating that stimulation did not selectively affect perception of, for example, the ‘below threshold’ voice stimuli.

**Table 2:** Hit rate (%) for the four different volume levels of voice embedded in the white noise; 0=overall false alarm rate (voice-absent trials).

Volume level	*M*	SD
4	98.6	3.67
3	66.6	17.53
2	44.5	15.88
1	22.5	12.60
0	10.6	9.86

A 3×2×2 (stimulation condition×task block×stimulation strength) mixed model ANOVA with response bias (*β*) as the dependent variable showed a main effect of stimulation condition, approaching significance (*F*(2, 56)=2.8, *p* =.069, *η*^2^ =.091). Planned contrasts (one-tailed paired samples *t*-tests) showed that *β* under the anodal stimulation condition was significantly higher than under the cathodal stimulation condition (*t*(29)=2.19, *p* =.019). (See Table 1 for descriptive statistics.) *β* under the anodal condition differed significantly from sham stimulation, (*t*(29=1.74, *p*=.046), but the sham condition did not differ from the cathodal condition (*t*(29) =.17, *p*=.43). There was also an effect of task block (*F*(1, 28)=21.74, *p*<.001, *η*^2^ =.437), suggesting that participants became less willing to respond that a voice was present with more experience of the task. There was no interaction between stimulation condition and task block (*F*(2, 56)=2.39, *p* =.101), or any interactions between any variables and stimulation strength (all *p*s >.18).

A 3×2×2 (stimulation condition×task block×stimulation strength) mixed model ANOVA with sensitivity (*d*′) as the dependent variable showed that there was a main effect of tDCS on task sensitivity approaching significance: *F*(2, 56)=3.17, *p*=.05. Planned contrasts (paired samples *t*-tests) showed that there was a significant difference between anodal and cathodal stimulation conditions (*t*(29)=2.79, *p* =.005), but no significant difference between anodal and sham conditions (*t*(29)=1.05, *p* =.30) or the sham and cathodal conditions (t(29)=1.25, *p* =.22). This difference between anodal and cathodal conditions can be accounted for by the aforementioned larger change in false alarm rate than hit rate, as an increased false alarm rate, but stable hit rate, will lead to a decrease in sensitivity to the task. (In other words, as false alarm rate increases, the difference between the hit rate and false alarm rate decreases, leading to a lower *d*′ score.)

## 4 Discussion

The present study used tDCS to study the effect of modulating cortical excitability of the left posterior superior temporal gyrus (pSTG) on rate of auditory false perceptions reported in white noise. The results showed that, as predicted, there were significantly more false alarm responses when excitability was increased in this region using anodal stimulation, than when excitability was decreased with cathodal stimulation. The false alarm rate in the sham stimulation condition lay at a mid-point between that observed for anodal and cathodal stimulation, with the comparison with sham non-significant in each case. Signal detection analysis revealed that changes in performance due to stimulation were related to both changes in response criterion (bias) and in task sensitivity. Our findings can thus be taken to demonstrate that the left pSTG plays a role in the generation of auditory false perceptions in a non-clinical population. This is consistent with neuroimaging results showing that false alarm responses on auditory signal detection tasks are associated with over-activation of the STG, above and beyond that seen when participants correctly report hearing a voice (Barkus et al., 2007), and crucially provides evidence that this cortical area is *causally* involved in false alarm responses.

In signal detection terminology, as a result of the stimulation condition we observed a change approaching significance in response bias, where participants were more willing to respond that a voice was present when stimulation to increase the excitability of pSTG was applied. Furthermore, there was a decrease in sensitivity under the anodal stimulation condition, and an increase in sensitivity after cathodal stimulation (indicating that increasing excitability made it more difficult for participants to distinguish between the voice signal and the noise, whereas decreasing excitability made it easier). Previous studies have found that individuals who experience auditory hallucinations show a lower response bias (*β*), but a similar level of sensitivity (*d*′) on auditory signal detection tasks, compared to non-hallucinating individuals (Brookwell et al., 2013), which has been taken as evidence that AVHs are associated with a bias towards labelling ambiguous percepts as external. These findings are also consistent with findings that hallucinating individuals show higher levels of activity in primary auditory cortex in response to internally generated processes such as AVHs (Kompus et al., 2011).

However, the present findings are only partially consistent with the hypothesis that differing levels of activation in the pSTG result in a differential response bias, since a change in task sensitivity was also observed as a result of stimulation condition. Nevertheless, some studies have shown a difference in sensitivity to signal detection between hallucinating and non-hallucinating groups (Vercammen et al., 2008), which may implicate broader differences in the way auditory verbal stimuli are processed in individuals prone to hallucinations. Our results suggest that higher levels of activity in the pSTG causes a bias to responding that a signal is present, but also make it more difficult to distinguish between signal and noise. Clearly, the stimulating electrodes are not simply creating an ‘analogue’ of individuals who experience AVHs; it is possible that, at a neural level, stimulation to increase excitability in pSTG could have reduced the signal-to-noise ratio, making it difficult to distinguish between internally and externally generated perceptions (as evidenced by the change in sensitivity).

The pSTG includes secondary auditory cortical areas such as Wernicke׳s area and the planum temporale (PT), as well as the temporoparietal junction (TPJ). The PT lies within secondary auditory cortex, and is preferentially active to auditory stimuli located in the external environment (Hunter et al., 2002). Thus, as well as lowering sensitivity, aberrant activation of this area may lead to a higher likelihood of a stimuli being attributed to an external source (as seen in the change in response bias). This is consistent with functional magnetic resonance imaging (fMRI) evidence showing that AVHs experienced as located in the external environment are associated with higher levels of activation in the PT (Looijestijn et al., 2013). The TPJ, meanwhile, has been implicated in feelings of ‘sensed presence’, and intracranial stimulation of this area can lead to disrupted self-processing (Blanke, Ortigue, Landis & Seeck, 2002). Resting state fMRI suggests that aberrant functional connectivity between TPJ and language production areas may be associated with AVHs (Vercammen, Knegtering, den Boer, Liemburg & Aleman, 2010) which may underlie problems monitoring self-produced (inner) speech. Taken together, these findings indicate that the TPJ and other posterior temporal regions may play a key role in distinguishing between self-generated and externally-generated perceptions.

However, the area stimulated in the current study (25 cm^2^) does not allow us to disentangle the potential roles of specific areas of the pSTG in this task. Indeed, a limitation of the technique employed in this study is the relatively low spatial resolution, and so it is not possible to test whether differences in levels of activation of, for example, the PT or TPJ, drive the observed effect, and it is also possible that the stimulating electrode could have affected inferior parietal regions of cortex. Whilst it is not possible to resolve this issue using the current data, it should be noted that, in a neuroimaging study using a very similar task, other cortical regions such as the inferior parietal lobe were not implicated in false alarm responses (Barkus et al., 2007). In principle, stimulating electrodes with a smaller surface area could be used to investigate the role of more specific cortical regions (Borckardt et al., 2012), although these techniques are still in their infancy, and the higher current density (due to smaller electrode surface) can lead to discomfort for the participant. A combination of noninvasive neurostimulation, such as the technique used in the present study, and functional neuroimaging techniques, would allow investigation of precise cortical areas involved in false alarm responses. This would also allow exploration of effects that may be distal to the stimulating electrode, and potentially part of a network of cortical areas involved in the genesis of auditory false perceptions.

It is, furthermore, possible that tDCS could affect functional interactions between pSTG and other regions of a cortical network thought to be involved in auditory false perceptions (for example, anterior cingulate regions involved in error detection or inferior frontal regions involved in speech production; Allen, Larøi, McGuire, and Aleman, 2008). Alternatively, Hoffman, Fernandez, Pittman and Hampson (2011) have argued that a corticostriatal loop involving Wernicke׳s area, Broca׳s area, and the putamen may be hyperconnected in those who experience AVHs, and that this may be affected by left TPJ stimulation. Again, integration of neurostimulation and neuroimaging paradigms will enable exploration of these issues (for a recent example, see Ellison et al., 2014).

We also observed a reduction in false alarm rate between the first and second task block, regardless of stimulation condition; that is, participants were less prone to auditory false perceptions in the offline block (after stimulation) regardless of whether they had received sham stimulation or either of the active stimulations. This is in accordance with the practice effect in auditory signal detection noted by Varese et al. (2011). There was no interaction between stimulation condition and task block, implying that the increased or decreased excitability of the pSTG was still evident in the offline block of the signal detection task. This is consistent with previous findings which have indicated that, after 15 min of stimulation, behavioural effects can be seen for up to an hour (Hummel & Cohen, 2006). Our results also indicated that decreasing the strength of the stimulation applied, from 1.5 mA to 1 mA, did not significantly affect the results (that is, there was an effect of stimulation on false alarm rate regardless of stimulation strength). It might be expected that, if the stimulation strength was decreased, a smaller effect size would be observed, but it is probable that the present study was not adequately powered to pick up an interaction between stimulation condition (anodal, cathodal or sham) and stimulation strength (1.5 mA/1 mA). Investigating this interaction was not, however, a primary aim of the study.

One possible alternative interpretation of these findings relates to the positioning of the ‘reference’ electrode above the right eye. This electrode likely covered anterior areas of the right prefrontal cortex, an area which has previously been implicated in source memory retrieval (Simons, Davis, Gilbert, Frith & Burgess, 2006). It is, therefore, possible that the reported effects are due to modulation of activity in this brain region; however, neuroimaging using auditory signal detection tasks did not specifically associate this brain area with any aspects of performance, whereas superior temporal regions were specifically associated with false alarm responses (Barkus et al., 2007). We also attempted to minimise the potential effect of the frontal electrode by increasing the size of the electrode, therefore decreasing the current density and lessening the potential for neuronal modulation. Whilst it seems more parsimonious to conclude that the observed effect was due to changes in activity of the pSTG, it cannot be ruled out that modulation of areas in prefrontal cortex may be responsible for the observed effect on auditory signal detection. Indeed, many other studies utilise a contralateral frontal electrode (e.g., Ball et al., 2013, You et al., 2011), and so this is an issue which pervades much tDCS research.

Regardless of limitations relating to spatial resolution, this study has provided evidence that stimulating using this electrode montage can affect the number of auditory false perceptions on a signal detection task. This has implications for the potential of using neurostimulation as a treatment option, with studies attempting to reduce activity in the posterior STG suggesting that this may be an efficacious treatment option to reduce their frequency (Slotema et al., 2014). It has recently been suggested that modulation of a cortical network important in self/reality monitoring and inner speech may underlie the therapeutic effect of neurostimulation (Moseley et al., 2013), although evidence concerning the effect of neurostimulation on reality monitoring is limited. This would, however, be consistent with the present results, and with neuroimaging findings relating to activity in the STG during source memory and signal detection tasks (Barkus et al., 2007, Sugimori et al., 2014), which imply that higher levels of activation may lead to perceptions being labelled as external. An interesting avenue for future research would be to investigate whether stimulation of the pSTG would affect response biases in detecting stimuli other than voices. It has been relatively well established that individuals that hallucinate are more likely to misattribute auditory verbal material (Brookwell et al., 2013), but less research has investigated other modalities. Gawęda, Woodward, Moritz, and Kokoszka (2013) found that patients with a diagnosis of schizophrenia who hallucinated showed a bias towards responding that imagined actions were actually performed, indicating that response biases may not be specific to voices, or, indeed, auditory stimuli.

Future research should therefore attempt to establish the precise relationship between reality monitoring (distinguishing between internally and externally generated perceptions) and the auditory signal detection task used here, as well as the specificity of the effect to auditory verbal material. Previous literature has tended to assume that the differential response bias shown by hallucinating participants in auditory signal detection is linked to reality monitoring mechanisms (Bentall and Slade, 1985, Brookwell et al., 2013). One could argue that a false alarm response must, by definition, be an internal mental event misattributed to an external source (i.e., the white noise), and this idea is supported by meta-analytic findings, which show a similar effect size between hallucinating and non-hallucinating samples for response biases on source memory tasks and auditory signal detection tasks (Brookwell et al., 2013). Here, we have shown that modulating excitability of left pSTG can alter the false alarm rate, and it is possible that this may be due to modulation of activity in areas important for reality monitoring, but it is not clear whether this finding would be specific to language. Future research should aim to establish whether neurostimulation of pSTG can have a similar effect on other tasks purported to test reality monitoring.

In conclusion, this study demonstrates that modulating activity in the pSTG can affect the number of false alarm responses that participants make when asked to detect speech in white noise. These results are consistent with theories that specify an important role for the pSTG in mechanisms that distinguish between internally and externally generated perceptions. It also provides a mechanism through which modulation of excitability of this cortical region may reduce frequency of AVHs, in those that seek help with anomalous experiences.

## Funding

This work was supported by the 10.13039/100004440Wellcome Trust (Grant number WT098455MA), as well as the Seedcorn Research Fund and Faculty of Science at 10.13039/501100001314Durham University, UK. The sponsors had no role in study design.
